# Draft Genome Sequence of a *bla*_KPC-2_-Carrying Citrobacter braakii Isolate from Pediatric Hospital Wastewater in Peru

**DOI:** 10.1128/MRA.00569-21

**Published:** 2021-07-29

**Authors:** Guillermo Salvatierra, Alejandra Dávila-Barclay, Brenda Ayzanoa, Diego Cuicapuza, Carlos Santillán-Salas, Pablo Tsukayama

**Affiliations:** a Laboratorio de Genómica Microbiana, Departamento de Ciencias Celulares y Moleculares, Facultad de Ciencias y Filosofía, Universidad Peruana Cayetano Heredia, Lima, Peru; b Instituto Nacional de Salud del Niño San Borja, Lima, Peru; c Instituto de Medicina Tropical “Alexander von Humboldt,” Universidad Peruana Cayetano Heredia, Lima, Peru; d Wellcome Sanger Institute, Hinxton, United Kingdom; University of Arizona

## Abstract

Here, we report a draft genome sequence of a *bla*_KPC-2_-carrying Citrobacter braakii isolate from pediatric hospital effluent. Genome CF248 represents a multidrug-resistant *C. braakii* isolate derived from a clinical environment in Peru.

## ANNOUNCEMENT

*Citrobacter* is a motile, facultative, anaerobic member of the *Enterobacteriaceae* associated with nosocomial infections (1, 2). The emergence of *Citrobacter* members as nosocomial pathogens causing urinary and respiratory tract infections has been described (3–6), along with reports of clinical isolates carrying horizontally transferred antibiotic resistance genes (ARGs), such as *bla*_KPC-2_ (7). Hospital water effluents may act as reservoirs for ARG-carrying bacteria, including carbapenemase-producing *Enterobacterales* (8). Routine monitoring of wastewater can detect resistant pathogens released into municipal wastewater and natural water bodies (8, 9).

We recovered isolate CF248 in December 2017 as part of a pilot program to monitor hospital effluent from a pediatric hospital in Lima, Peru. The precipitate of a 50-ml water sample collected from an exit sewage pipe was swabbed onto MacConkey agar (Becton, Dickinson, Heidelberg, Germany) and incubated for 24 h at 37°C. One lactose-fermenting colony was restreaked onto MacConkey agar and classified as a *Citrobacter* sp. with the Phoenix automated microbiology system (BD Diagnostic Systems, Sparks, MD, USA). We identified resistance to 11 different antibiotics, including ertapenem, imipenem, and meropenem (Table 1), following CLSI guidelines (10). KPC production was confirmed with the RESIST-4 OKNV (Coris BioConcept, Gembloux, Belgium) immunochromatographic rapid diagnostic test for carbapenemase detection.

**TABLE 1:** Antimicrobial susceptibility testing of the Citrobacter braakii CF248 isolate

Antibiotic	MIC (μg/ml)	Interpretation^*a*^
Aminoglycosides		
Amikacin	≤8	S
Gentamicin	≤2	S
Penicillins		
Ampicillin	>16	R
Penicillins + β-lactamase inhibitor		
Ampicillin-sulbactam	>16/8	R
Piperacillin-tazobactam	>64/4	R
Cephalosporins		
Cefazolin	>8	R
Cefepime	8	I
Cefoxitin	>16	R
Ceftazidime	16	I
Ceftriaxone	>4	R
Fluoroquinolones		
Ciprofloxacin	>2	R
Levofloxacin	>4	R
Sulfonamides		
Trimethoprim-sulfamethoxazole	≤0.5/9.5	S
Glycylcyclines		
Tigecycline	2	S
Carbapenems		
Ertapenem	>1	R
Imipenem	8	R
Meropenem	8	R

a S, susceptible; I, intermediate; R, resistant.

Genomic DNA was extracted from 1 ml of tryptic soy broth (Becton, Dickinson) single-colony culture incubated at 37°C for 6 hours, using the GeneJET genomic DNA purification kit (Thermo Fisher Scientific, Waltham, MA, USA). Nextera XT libraries (Illumina, Inc., San Diego, CA, USA) were sequenced using a MiSeq instrument generating 1,735,120 paired-end 250-bp reads. Reads were processed and analyzed for ARG detection with the tools within the Nullarbor v.2.0 pipeline (11), using SPAdes (v.3.15.2) (12) and Prokka (v.1.13.3) (13) for assembly and annotation, respectively, resulting in a draft genome of 294 contigs larger than 300 bp (*N*_50_, 49,265 bp) and a total length of 5,810,531 bp, with a 64× mean coverage and a GC content of 52.3%. Plasmid replicon typing was performed using plasmidfinder v.2.0 (14), which is available at the Center of Genomic Epidemiology (www.genomicepidemiology.org). Antibiotic resistance genes and plasmid replicons with a cutoff value of >95% for the identity and a coverage of 100% were included. Default parameters were used for all software tools.

Resistome analysis identified ARGs for aminoglycosides [*aph(3')-VIa*], fluoroquinolones (*qnrB68*), and β-lactams (*bla*_CMY-83_ and *bla*_KPC-2_). We identified plasmids of incompatibility (Inc) groups HI1A (CIT), HI1B (CIT), FIB (K), and P6. The plasmid replicon IncP6 has been reported as a carrier of *bla*_KPC-2_ in Pseudomonas aeruginosa (15), Klebsiella oxytoca, Enterobacter cloacae, Citrobacter freundii (16), and Citrobacter braakii (17). Contig assessment revealed that the *bla*_KPC-2_ gene was located on a Tn*3* transposon element of 13.8 kb with the linear structure IS*Kpn27*-Δ*bla*_TEM-1_-*bla*_KPC-2_-IS*Kpn6*-*korC-klcA-repB*. BLAST analysis of the *bla*_KPC-2_ gene sequence showed 100% sequence identity and 100% query coverage with the 111,195-bp plasmid pKPHS2 (accession number NC_016846) isolated from a clinical *bla*_KPC-2_-carrying Klebsiella pneumoniae isolate in China.

Here, we report a *bla*_KPC-2_-producing *C. braakii* isolate derived from hospital wastewater in Peru. Our results suggest that antibiotic resistance monitoring of hospital wastewater may detect antimicrobial resistance (AMR) dissemination from clinical settings into the sewage environment.

### Data availability.

The draft genome assembly of Citrobacter braakii CF248 has been submitted to NCBI under accession number GCA_018449455.1 under BioProject PRJNA729854, and the raw reads have been submitted to the SRA under accession number SRR14534310.
